# Maximum longevity and juvenile mortality in zoo‐housed mangabeys

**DOI:** 10.1002/zoo.21690

**Published:** 2022-04-01

**Authors:** Manon de Visser, Emile Prins, Mirte Bosse, Richard Crooijmans, Tjerk ter Meulen

**Affiliations:** ^1^ Wageningen University & Research, Animal Breeding and Genomics Wageningen The Netherlands; ^2^ GaiaZOO Kerkrade The Netherlands; ^3^ Present address: Evolution & Biodiversity, Institute of Biology Leiden Leiden University Leiden The Netherlands; ^4^ Evolution & Biodiversity, Institute of Biology Leiden Leiden University Leiden The Netherlands; ^5^ Naturalis Biodiversity Center Leiden The Netherlands

**Keywords:** demographics, infant mortality, lifespan, parity, primate

## Abstract

Little is known about the biology of grey‐cheeked and black crested mangabeys (*Lophocebus albigena* and *Lophocebus aterrimus*, respectively). As these primates face threats in the wild, well‐monitored zoo‐housed populations with up to date registries are becoming increasingly valuable to acquire species knowledge and to support conservation efforts. We used international studbooks to extract demographic and genetic information on 519 mangabeys to investigate how life history and parent‐related variables influence maximum longevity and juvenile mortality. Generalized linear mixed models, as well as survival analyses, were applied. Results showed that females lived significantly longer than males, which is not uncommon in primates. Furthermore, our results indicated that the maximum longevity is lower for individuals living in European zoos versus individuals from North American zoos, which may be due to a combination of environmental differences and potential founder effects. We also show that the maternal maximum longevity is positively related to the maximum longevity of the offspring, which may be explained by the inheritance of “good genes“. However, the age of the mother at the moment of birth was negatively related to the maximum longevity of the offspring, which contradicts literature that states that, in primates, more experienced and thus older mothers will raise their offspring better than less experienced mothers. Instead, it is more likely that an “optimal age range” exists for breeding mothers. Our study provides insights into the population biology of captive mangabeys and may be helpful for identifying future research priorities to optimize primate health and welfare directly ex situ, and indirectly in situ.

## Research highlights

In general, females grow older than males. Maternal, age‐related factors can serve as a proxy for longevity, including juvenile mortality rates, in offspring. Also, an optimal breeding age range for mothers is likely. An intercontinental difference in overall lifespans was observed.

## Graphical abstract

The international studbooks of two *Lophocebus* mangabey species were analyzed. Sex, as well as traits of the mother, serve as proxies for the maximum longevity of individuals. Also, intercontinental differences in cumulative survival rates seem to exist for the captive populations of these mangabeys; underlying effects remain to be discovered.

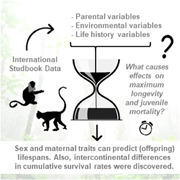

## 1 INTRODUCTION

Anthropogenic forces are the main reason many animal species are rapidly declining (Wagler, 2011). Zoo‐housed populations are becoming increasingly important as zoos progressively support captive breeding and reintroduction programs for species conservation (Braverman, 2014; Conde et al., 2013; Frankham et al., 2004). Furthermore, the zoo industry has seen rapid improvements in the optimization of the health and welfare of the animals involved over the past few decades (Kagan et al., 2015; Kawata, 2009; Leus & Lacy, 2011; Whitham & Wielebnowski, 2013). To provide the best possible care and housing, it is desired to know as much as possible about the natural population dynamics and the biology of the species in question. Unfortunately, for many animal species, this type of information is currently still limited and it would require long‐term field studies to obtain it. When such studies are lacking, or difficult to establish and pursue, records from animals housed in zoos provide an alternative (Conde et al., 2019; Princée, 2016).

The black crested mangabey (*Lophocebus aterrimus*) and the grey‐cheeked mangabey (*Lophocebus albigena*) are examples of primate species with a relatively low amount of documented field studies in wild populations, but with good quality studbook data available on captive populations. The studbooks of these mangabey species have been managed by zoos for over five decades. Studbooks are digital databases with records that cover the pedigree and life history information of zoo populations (Frankham et al., 2004; The Zoological Society of London, 2016). Besides genetic information, data on demographic parameters, such as births and deaths, can be obtained from zoological studbooks (Frankham et al., 2004; Leus & Lacy, 2009).

The two species under study are considered closely related and they occur naturally in Central Africa, where the black crested mangabey is known to live south of, and the grey‐cheeked mangabey north of, the Congo River (Burrell et al., 2009; Gautier‐Hion et al., 1999; Patel, 2005; Singleton, 2009; Tosi et al., 2003). For both species continuing population declines are currently recognized as a result of illegal hunting and habitat loss (Gautier‐Hion et al., 1999). Since the global reassessments of 2017 both species are considered “Vulnerable” by the IUCN Red List of Threatened Species with ongoing, rapid population declines (Maisels, Hart, & Laudisoit, 2020;).

The decrease in numbers is alarming. Firstly, these species are mostly frugivorous and are therefore identified as important small‐bodied seed dispersers within their habitat (Babweteera & Brown, 2010; Beaune et al., 2013; Gautier‐Hion & Maisels, 1994; Horn, 1987; McGraw, 1994; Poulsen et al., 2001; Shah, 2003; Trolliet et al., 2016). Secondly, as relatively large‐bodied mammals are quickly hunted out and the remaining “empty forests” are subjected to intensifying logging practices, hunters shift the focus of their poaching activities towards medium‐ and small‐bodied animals as a result of “supply and demand” (Maisels, Hart, & Laudisoit, 2020; Maisels, Hart & Olupot, & Oates, 2020). This means that any further decline of these primates also indirectly creates higher risks for other animals that inhabit the same areas, and for the forests themselves.

There is a need to study and protect these primates. By saving the mangabeys from extinction, eco‐tourism and thereby local communities would benefit (Wich & Marshall, 2016). The conservation of these species may also promote the conservation of other taxa, by functioning as inadvertent flagship species. This is because humans identify themselves the most with animals that are the most similar to them, which increases their urges to help and safeguard nature (Chan, 2012; Meyers Jr et al., 2009; Wich & Marshall, 2016). Furthermore, any knowledge acquired on mangabey biology can ultimately be of relevance in the context of human health in and it also provides insights into overall primate evolution (Wich & Marshall, 2016). Lastly, to comply with animal welfare standards that are nowadays commonplace in modern zoos, research toward “environment‐based” and “animal‐based” factors that may influence the health of captive animals is officially required. The importance of understanding the overall biology of zoo‐housed animals should not be underestimated (EAZA, 2021).

The aim of this study is to investigate the effects of life history variables and parental variables on the maximum longevity and on juvenile mortality rates in captive populations of grey‐cheeked and black crested mangabeys. We focus on maximum longevity, because it is an often used welfare and health‐related indicator that is also easy to measure, as it requires merely a birth and death date (Broom, 1991; Veasey, 2017). Juvenile mortality rates are analyzed as well, but separately, as it is known that primates in captivity lose relatively many premature births compared to other animal groups (Kohler et al., 2006; Young & Heard‐Booth, 2016). This is because various life history and environmental factors can influence both the breeding success of captive primate mothers, as well as the survival chances among infants and juveniles (Debyser, 1995; Levallois & De Marigny, 2015; Saiyed et al., 2018; Schwitzer & Kaumanns, 2009).

To the best of our knowledge, our study provides a first insight into the demography of zoo‐housed *Lophocebus* mangabeys from European and North American zoo populations. By investigating the international studbooks (ISBs), we can shed the first light on the overall population health and biology. This will help optimize current management practices of these, and perhaps also other, primate species kept within the zoo community and may ultimately support in situ nature management as well.

## 2 MATERIALS AND METHODS

### 2.1 Studbook data extraction

Data were extracted from the International Studbooks of the black crested mangabey (version May 2017) and the grey‐cheeked mangabey (version April 2017), which are coordinated by GaiaZOO, the Netherlands. These studbook versions were most recently validated for scientific use at the same moment in time. The ISBs contain all available data of animals that are, or were once, held by: (1) member institutions of the Association of Zoos and Aquariums (AZA); (2) member institutions of the European Association of Zoos and Aquaria (EAZA); or (3) nonaccredited zoos that collaborated with either AZA, EAZA, or both associations.

Our focus lies on the entire historic and current population. This means we maximized the demographic export window and used all the information from the first records until the last updates recorded in spring 2017. The first recorded, estimated birth date of a wild‐caught individual black crested mangabey stemmed from the year 1934. For the grey‐cheeked mangabey, this was 1953. According to anecdotal evidence, it was mostly the infants and juveniles that were considered suitable for trade, and thus for the establishment of captive populations, in the zoo community in the past. The age of wild‐caught animals was believed to be estimated shortly after capture and was based on, inter alia, body size. In the records, birth dates were generally set to the first day of the estimated birth year. Over more recent decades, modern zoos generally have not taken in wild‐born individuals, as efforts are now focused on conserving wild populations and reintroducing wild‐caught individuals into their native habitat as much as possible. Thus, birth date estimations apply primarily to historic population data.

We investigated the data on genus level (*Lophocebus*), rather than species level, after we checked whether measures of maximum longevity were indeed comparable. We then combined black crested and grey‐cheeked mangabey data, assuming similar trends in biology and demography due to the close relatedness of the two species. By making this choice, we maximized our sample size and thus the power of our study. Studbook variables (Table 1) were extracted from Species360 software SPARKS Version 1.66 exported reports (Scobie & Bingaman Lackey, 1997; Scobie & Flesness, 1989). In addition, the software program PMx Version 1.4.3 (Lacy et al., 2012) was used to calculate genetic variables directly from the pedigrees, such as the inbreeding coefficient (*F*‐adjusted).

**Table 1:** Overview of all the variables that are investigated in the generalized linear mixed models (GLMMs)

Variable	Definition
Dependent variables	
Maximum longevity	Maximum age in days (until death).
Juvenile mortality	Death under certain infant age thresholds (1 day, 1 year, or 3 years).
Independent variables	
Dead‐lost‐alive	The status of the animal (“dead“, “lost to follow up (LTF)“, or “alive”).
Sex	The sex of the according individual (male, female, or unknown).
Birth type	Whether the animal was captured, or born in a zoo.
Rearing	The way the individual was reared as an infant (parent‐reared vs. otherwise).
Inbreeding	The inbreeding coefficient per individual (*F*‐adjusted as calculated by PMx).
Father ID	The identity of the father.
Father age at offspring birth	Age of the father when the individual was born in days.
Mother ID	The identity of the mother.
Mother age at offspring birth	Age of the mother when the individual was born in days.
Mother parity	Whether the mother has had offspring before (either recorded as being primi‐ vs. multiparous, or as an exact number of previous offspring, depending on the model).
Maternal maximum longevity	Maximum age of the mother in days (until death).
Rearing type mother	The way the mother of the individual was reared (parent‐reared vs. otherwise).
Region born	The region/continent in which the animal was born (Europe, North America, or Africa).
Region familiar	The region/continent the animal has lived in for the most time of its life (Europe, North America, or Africa).
Zoo born	The zoo in which the animal was born.
Zoo familiar	The zoo in which the animal has lived for the most part of its life.

*Note*: A definition is given per variable. If the data were unavailable or unknown for certain individuals, no data were entered. Further explanations regarding these definitions and/or the extraction methods, as well as more information on descriptive statistics, can be found in Table SI.

In our study, we use the age of three as a clear threshold for deceasing as a youngster (=“juvenile mortality”) versus deceasing as an adult. The period between being born and reaching the age of three comprises of different life stages, ranging from infant, to juvenile to (pre‐)puberal, but not yet fertile. We categorized juvenile mortality into three distinct classes: death within one day (including miscarriages), death within the first year, or death within three years. The first year is considered the most critical in terms of survival, as mangabeys start weaning after two months and become increasingly independent of the mother when the age of one is reached (Abelló et al., 2018).

We always took into account whether individuals were still alive according to the studbook, or whether they had indeed deceased. The cause of death, or whether euthanasia was performed, was unfortunately inconsistently documented in the studbooks. In addition, incidents such as accidents or deadly fights may have occurred, but they are considered rare. Our study is thus based on the assumption that overall, in adult individuals, deaths occurred due to poor health or old age (including the cases that required euthanasia). The usage of contraceptives was also not taken into account in the analyses that included paternal information, as this was not documented in detail and is also strongly time‐dependent.

### 2.2 Data preparation and analysis

To figure out which independent variables had an effect on maximum longevity or on juvenile mortality, we applied survival analyses (SA, both Kaplan‐Meier and Cox Regression) and built generalized linear mixed models (GLMMs) using IBM SPSS v.22.0 (IBM Corp, 2013). Survival analyses are highly useful because they can incorporate information of both deceased as well as alive individuals by analyzing the occurrence of “events” (in our case, this “event” is the recorded moment of death). GLMMs are relatively flexible and these types of models allow for the investigation of a target with non‐normal distribution, provide the application of a suitable link function, can handle missing data, and can include random effects (Bolker et al., 2009). The variables investigated in the study are summarized in Table 1 and a more elaborate version of this table is provided in Table SI.

With the Kaplan–Meier survival analyses we calculated cumulative survival curves to compare maximum longevity in adults between the sexes, between birth continents (“region born”), and between the continents where the animal had lived at for most of their lives (“region familiar”). Animals that originated from Africa were, in our case, by definition born in the wild.

The influence of parental parameters on maximum longevity in adults was explored using a GLMM (Model A). Another GLMM focused on juvenile mortality (Model B) and included both parental information as well as life history traits. The models were based on a robust estimation of covariance to handle any possible violations of model assumptions. By comparing the Akaike Information Criterion (AIC) scores between different models and by using a backward, step‐wise elimination method, minimum adequate models (MAMs) were built originating from full models (FMs).

In Model A, only adult animals (i.e., individuals that reached an age of three years and older) were considered for input. We include both deceased and alive individuals and corrected for this “vital status” of the animals by introducing the random factor “dead‐lost‐alive” (Table 1). The identity of the mother and, if known, the identity of the father, was corrected for as well by including them as random factors, as some individuals shared either one or both parents, for instance. For maternal as well as paternal information, the following variables were included in the FM, with main and two‐way effects: “mother age at offspring birth“, “rearing type mother“, “maternal maximum longevity“, “mother parity” (being either primi‐, or multiparous) and “father age at offspring birth”, in case the latter was known. Paternal information was generally more limited and thus we decided to not further risk a reduced sample size based on this scarce information. Cox Regression models were additionally used to predict the maximum longevity based on the combined effects of the maternal maximum longevity and the age of the mother at offspring birth.

In Model B, only deceased animals belonging to either one of the three infant age classes were included. Again, the mother's and, if known, the father's identities were included as random factors. Also, we included “region born” as a random factor here to correct for young animals being wild‐caught or not, as well as to correct for any continental differences. We were more interested in other variables for this model, and thus included in the FM the following factors with main effects: “sex“, “inbred“, “zoo born“, “mother age at offspring birth“, “maternal maximum longevity“, “mother parity” (in this case, the recorded number of previous offspring), and “father age at offspring birth“, in case the latter was known.

A Gamma distribution and log‐link function were applied for Model A. Because of the ordinality of the target variable, a Multinomial logistic regression with cumulative logit function was applied in Model B. Before building the models and deciding which variables to include, we checked the degree of multi‐collinearity between explanatory variables and avoided the uptake of variables that correlated significantly. We used the Variance Inflation Factor (VIF) value of 10, which indicates a high correlation, as a strict threshold for FMs (Alin, 2010; Eisenlohr, 2014; Olivia & Ilie, 2013). However, we made sure that the final MAMs only included variables that showed VIF values of a more conservative threshold of 2.5.

## 3 RESULTS

Including both the current and the historic populations, the total number of individuals of black crested mangabeys equaled 363 (178 males, 167 females, and 18 of unidentified sex) and the total number of grey‐cheeked mangabeys was 156 (71 males, 74 females, and 11 of unidentified sex). Overall, the recorded animals had together been associated with over 97 different institutions over time, which were almost exclusively located in either North America or Europe. Out of these 97 institutions, 29 held either one or two of the study species at the time the ISBs used for the analyses were last updated. In total, the dataset thus comprised of information on 519 individual mangabeys. Out of those, 98 were still alive when this study was performed. Of the rest, 352 deaths were recorded and the remaining 69 animals were “lost to follow up“, meaning that no death date is known.

Before any downstream analyses were conducted, we checked whether the maximum longevity observed in black crested and grey‐cheeked mangabeys was indeed comparable (excluding cases of juvenile mortality). We found no significant difference in the maximum longevity of both species (Mann–Whitney U, *L. aterrimus n* = 145, *L. albigena n* = 55, *p* = .760, see Figure SI). *L. aterrimus* showed a mean maximum longevity of 5636.8d (which is roughly 15.4 y, SE = 255.9d) with a median of 5195.0d (≈14.2 y). *L. albigena* showed a mean maximum longevity of 5766.5d (≈15.8 y, SE = 429.5d) and a median of 5890.0d (≈16.1 y).

When all categories of juvenile mortality were, however, included in this species comparison, a significant difference was found (Mann–Whitney U, *L. aterrimus n* = 225, *L. albigena n* = 127, *p* = 0.005), with *L. aterrimus* having a mean maximum longevity of 3,667.9d (≈10.0 y, SE = 242.0d) and median of 2,813.0d (≈7.7 y), compared to *L. albigena* with a mean of 2549.0d (≈7.0 y, SE = 311.8d) and median of 197.0d (≈0.5 y). For the *L. albigena* dataset this was based on 74 juvenile mortality cases out of a total of 127 deaths, which equals 58.3%. For the *L. aterrimus* dataset this number was slightly lower with 96 juvenile mortality cases out of a total of 225 cases, equaling 42.7%. Thus, the maximum longevity of adults and juvenile mortality rates in infants were studied separately throughout the study.

For the downstream analyses, we let the program SPSS decide whether cases would be considered for processing in a certain model or not. This depended mostly on missing data, however, we aimed to include as many individuals as possible within all analyses. The results of the MAMs are provided in Table SII and are described below, together with the survival analyses results.

### 3.1 Survival analyses and the impact of the individual life history

We decided to investigate the effects of life history variables, such as “sex“, “birth type“, “region born“, and “region familiar“, on the maximum longevity by performing Kaplan‐Meier survival analyses. We excluded juvenile mortality cases. The effect of sex was investigated for all animals with a recorded sex (male: *n* = 206, female: *n* = 216). Cumulative survival appeared to be generally higher for females than for males (Kaplan–Meier, log‐rank: *χ* ^2^ = 7.184, *df*=1, *p* = .007, Breslow: *χ* ^2^ = 8.436, *df*=1, *p* = .004), with males having a mean maximum longevity of 4253.9d (≈11.7 y, SE = 302.7d) and a median of 3011.0d (≈8.2 y, SE = 497.0d) as opposed to females with a mean of 5599.4d (≈15.3 y, SE = 336.8d) and a median of 5292.0d (≈14.5 y, SE = 496.5d).

**Figure 1:**
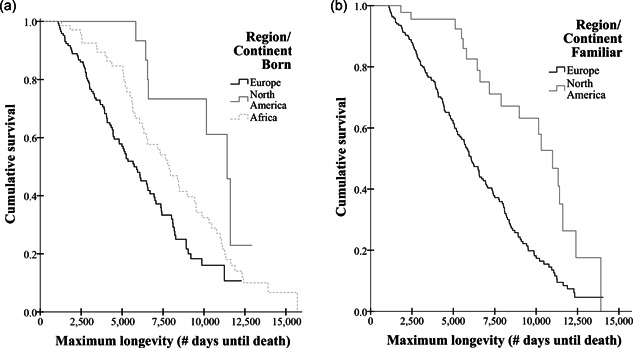
The cumulative survival depicted as calculated in the survival analyses, looking at birth region (a) and “familiar” region (b). In both graphs, juvenile mortality cases were excluded, which is why it seems as if the cumulative survival is equal to “one” in the initial stages of life (which is not the case in reality). In (b), only the animals that lived most of their lives in either European or North American zoos were included. In both graphs, the difference between European and North American zoos is apparent.

When focusing on birth type, a sample of *n* = 254 individuals was considered (of which 67 were wild‐born and 187 were zoo‐born). We found no significant difference in maximum longevity of wild‐born versus zoo‐born mangabeys (Kaplan–Meier, log‐rank: *χ* ^2^ = 2.651, *df*=1, *p* = .103). The test on birth region (“region born”) revealed a significant difference in cumulative survival between the three birth regions (Figure 1A, Kaplan–Meier, log‐rank: *χ* ^2^ = 19.410, *df*=2, *p* < .001), Europe (European zoos, *n* = 145, mean estimate = 6209.7d ≈ 17.0 y, with a median of 5736.0d ≈ 15.7 y), North America (North American zoos, *n* = 23, mean estimate = 10,307.0d ≈ 28.2 y, with a median of 11,414.0d ≈ 31.3 y), and Africa (wild habitat, *n* = 69, mean estimate = 8192.2d ≈ 22.4 y, with a median of 7884.0d ≈ 21.6 y). Furthermore, when focusing on “region familiar“, we also found a significant difference in cumulative survival of animals that lived most of their lives in European zoos (*n* = 224, mean estimate = 6526.6d ≈ 17.9 y, with a median of 6082.0d ≈ 16.7 y) versus North American zoos (*n* = 52, mean estimate = 9852.6d ≈ 30.0 y, median=10,991.0d ≈ 30.1 y) (Figure 1B, Kaplan–Meier, log‐rank: *χ* ^2^ = 18.114, *df*=1, *p* < .001).

### 3.2 The influence of parental factors on maximum longevity and juvenile mortality

**Figure 2:**
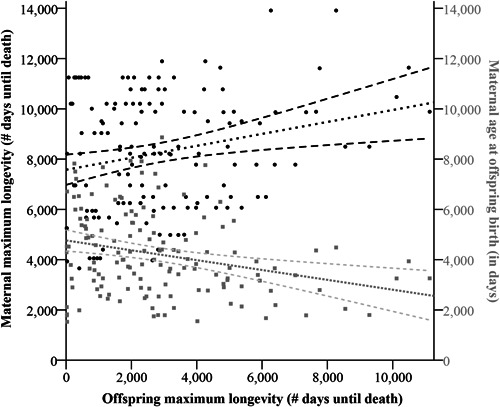
Relationships between the maximum longevity and maternal parameters. On the *y*‐axes, the maternal parameters are shown so that the significant effects of the maternal maximum longevity and the age of the mother at offspring birth as discovered in Model A become visualized. Note that maximum longevity, the dependent variable, is here depicted on the *x*‐axis and all ages are included. As the maximum longevity of the mother increases, so does the maximum longevity of the individual itself. As the age of the mother at the moment of offspring birth increases, the maximum longevity of the individual tends to decrease. The trend lines with 95% confidence intervals are given.

The MAM of Model A (VIF factors < 1.3) was based on 167 cases that could be considered for processing. In this model, we found significant main effects of “maternal maximum longevity” (*F* = 21.249, *p* < .001) and “mother age at offspring birth” (*F* = 47.811, *p* < .001). Within the MAM, the maternal maximum longevity positively influenced the maximum longevity of the offspring, whereas the age of the mother at the moment an individual offspring was born negatively influenced the maximum longevity of said offspring (Figure 2; Table SII). We found no significant effect of the rearing type of the mother on the maximum longevity of offspring; however, the number of animals reared in a way other than by their own parent was low in our dataset (19 animals in total, of which only six became mothers). We also discovered no significant effect of the paternal maximum longevity on the maximum longevity of the offspring.

As in Model A, both deceased and alive individuals were included in the Cox Regression analyses, but only in case the mother had already deceased (because here we were unable to correct for the vital status of the mother). In total 127 cases were included, with a significant interaction effect (Cox Regression: *χ* ^2^ = 6.741, *df* = 1, *p* = .009) between the maternal maximum longevity and the age of the mother at offspring birth when this interaction effect was investigated separately. We discovered no significant effects in a combined model that included both the main factors and the interaction factor, nor did we find significant main effects in separate models including only one, or both, of the main factors. These results are partially in line with the findings of Model A, although Model A brought forward significant main effects, whereas the Cox Regression models showed a significant cross‐over interaction.

Of the entire population, 170 individuals died as juveniles under the age of three years (81 were male, 60 were female, and the remaining individuals were unsexed). There is no significant difference in juvenile deaths between the two sexes (*χ* ^2^ = 3.145, *p* = .077). In total, 68 individuals died immediately the first day, 74 individuals died after day one, but still in their first year, and 28 individuals died after the first year, but before the age of three.

When focusing on juvenile mortality in Model B (VIF factors ≤ 2.5), a MAM was generated in which a total of 121 cases were considered for processing. We discovered that juveniles died sooner as: (1) the maternal maximum longevity decreased (*F* = 2.925, *p* = .090); (2) the age of the mother at offspring birth increased (*F* = 5.317, *p* = .023); and (3) when the mother had produced fewer offspring before (*F* = 5.745, *p* = .018; Table SII). Also here we found no significant effect of the paternal maximum longevity.

## 4 DISCUSSION

The aim of this study was to explore maximum longevity and juvenile mortality rates in zoo‐housed grey‐cheeked and black crested mangabeys. It can be difficult to work with rather small sample sizes from uncontrolled environments, especially when applying multivariate statistics (Tabachnick et al., 2007). Although such challenges are common in zoo research (Kuhar, 2006), we were able to discover interesting patterns regarding the impact of life history and parental factors on the maximum longevity and juvenile mortality rates in captive *Lophocebus* mangabeys.

### 4.1 Continental differences in maximum longevity

The outcome of the survival analyses shed a light on both life history and biological factors that predicted maximum longevity the most in our dataset. It should be again noted that birth dates of wild‐born individuals are generally rough estimates, while the birth dates of zoo‐born individuals are more accurate. However, considering the “preferred young age” of wild‐caught founders in the past, we assume that birth estimates of the historic population must be reasonable, and thus our findings plausible. We found no overall distinction between the maximum longevity for “birth type”: it seemed that both zoo‐born and wild‐born animals aged equally. This is something that has been observed in various species of zoo animal, such as in the naked mole rat (Novikov & Burda, 2013) and several deer species (Müller et al., 2010), likely because the benefits and disadvantages to being either in a wild or captive environment can balance each other out.

When no such balance exists, the maximum longevity between wild and captive animals will differ. It is for example known that some animals can thrive in zoo environments because of the lack of predation or parasitic pressures, as is the case for example in rodents and lemurs, respectively (Alexander et al., 2016; Ichino et al., 2015; Novikov & Burda, 2013). More examples of zoo‐thriving primate species include the rhesus macaque and gelada baboon (Tidière et al., 2016). On the other hand, it is known for some zoo‐housed animals that their wild counterparts generally live longer, as is true in elephants (Veasey, 2006; Wiese & Willis, 2004), as well as in some primates like chimpanzees, blue monkeys, and capuchins (Tidière et al., 2016).

Our results suggested that there are regional differences in aging. The adult mangabeys from North American zoos showed an overall higher cumulative survival than those from European zoos. This is most likely the result of environmental conditions that somehow differ between these two zoo environments and that directly, or indirectly, influence maximum longevity. Although literature on the maximum longevity between zoos from different continents is scarce, some studies do show that there can indeed be a difference. For example, in elephant populations, an opposite pattern was discovered with adult elephants in North American zoos living slightly shorter than elephants in European zoos (Wiese & Willis, 2004). Also, several studies exist for a variety of species that show substantial differences in housing and husbandry practices that likely impact animal welfare and longevity when comparing zoos in different, but also within the same, continents (Rose & Roffe, 2013; Rowden & Rose, 2016; Tanaka & Ogura, 2018). Of course, another reasonable explanation may be a difference in the genetic health of both populations due to, for instance, potential founder effects (Frankham et al., 2004).

### 4.2 Sex, as well as maternal traits, can predict longevity

The females live on average longer than males according to the outcome of the survival analyses. This is not uncommon in primates (Bronikowski et al., 2011; Gambini et al., 2005). Although we discovered no effect of the rearing type of the mother on the maximum longevity of offspring, it is known from studies in several mammal species that being hand‐reared, for instance, generally does not have a positive effect on the short and long‐term health of the animal in question (Hampson & Schwitzer, 2016; Maki et al., 1993; Porton & Niebruegge, 2006; Ryan et al., 2002). However, most of the studies performed on the effects of hand‐rearing versus parent‐rearing show negative impacts on reproduction and disturbed (social) behavior of the animals later in life, but not necessarily on the long‐term survival itself. They do show that, generally, juvenile mortality rates are higher with hand‐rearing, something we were unable to investigate through Model B because of the low number of cases that described this scenario.

For both Model A and Model B, a mild correlation between the maternal variables may have influenced the outcome of the models. Still, the VIF factors were acceptably low (≤2.5), meaning that the outcome of these models is not to be neglected. It is not surprising that maternal factors significantly influenced offspring lifespans. The positive effect of the maximum longevity of the mother on an offspring's own maximum longevity is also found in humans (Kemkes‐Grottenthaler, 2004) and evidence shows that there is a heritable component to longevity and individual fitness in mammals (Logan et al., 2016). The most recent estimates of research on humans suggest that “lifespan heritability” has been overestimated in the past due to assortative mating. However, in those recent estimations, a low heritability value nonetheless remained when correcting for the effect of assortative mating (Logan et al., 2016). It also has been described for a variety of mammalian zoo populations that the heritability of aging ranged between 0.17 and 0.53 (Ricklefs & Cadena, 2008). Thus, genetics could play at least a moderate role in our mangabey case, but it would require genetic studies to find out.

We did not discover any effect of the parity of the mother on maximum longevity of offspring in our adult‐focused Model A; however, we did discover an effect of maternal parity in the infant‐focused Model B. Conventionally, it is also believed that, in primates, more experienced, older mothers will raise more viable and successful offspring as opposed to less experienced, younger mothers, although it is not true in all primates (Pusey, 2012). Evidence is actually showing that offspring of primiparous mothers do not necessarily experience greater juvenile mortality (Nuñez et al., 2016). While it has been shown in wild populations of grey‐cheeked mangabeys that multiparous mothers had a higher breeding success (Arlet et al., 2014), and while our results suggested that deceased juveniles indeed had died sooner when the mother had had less offspring before, Model B focused only on mangabeys that died young anyway. Additionally, we found counterintuitive age‐related effects in both Model A and Model B.

Mothers that were older when giving birth seemed to reproduce viable offspring, even though the maximum longevity of the mother positively correlated with an individual's own maximum longevity and even though it can be assumed that, in general, older mothers must have had more offspring before in life, compared to younger mothers. Thus, an alternative scenario may apply in which an optimal breeding age range exists, which is the most likely considering the significant cross‐over effect we also discovered. Experience plays an embedded role and is not mutually exclusive.

An optimal age range has also been described in for instance ruffed lemurs (Tidière et al., 2018). It is caused by a natural reduction in reproductive capacity as individuals age, something that is called “reproductive senescence“, and it is known to influence the neonatal health and the survival of offspring negatively (Ahsan, 2014; Ericsson et al., 2001; Sharp & Clutton‐Brock, 2010; Vaughan et al., 2014). Additional mechanisms may be involved. In humans, for instance, older maternal age at offspring birth has been shown to have a significant negative effect on the maximum longevity of children in human families (Gavrilov & Gavrilova, 2015; Kemkes‐Grottenthaler, 2004), which is explained by the earlier maternal “mental loss” among children of older mothers (Myrskylä & Fenelon, 2012).

In our data, the few exceptions of cases where mothers gave birth at relatively old ages to viable offspring that became old themselves, may better be explained either by the heritable component that is associated with longevity. Still, the random effect of the mother's identity should have allowed the models to distinguish between the successes of relatively old versus relatively young mothers and also their parity. The potential influence of the emotional bond with the mother may complicate the matter, as well as explain why we only found maternal, not paternal, age‐related effects in both the adult‐ and infant‐focused models.

### 4.3 Recommendations for future research

In this study, we made use of the most recently validated ISBs of similar validation moments (spring 2017). We are aware that this means we are missing four to five years of data gathered under modern husbandry practices, which likely differ from those of the more distant past. Many zoos that currently hold either one or both study species have been connected to EAZA or AZA for years now. These associations and their member institutions have all adopted similar policies and procedures that are in accordance with guidelines by the World Association of Zoos and Aquariums, also: the “umbrella” organization (Dick & Gusset, 2010; Whitham & Wielebnowski, 2013).

The animals included in our study were being managed by both non‐accredited and accredited institutions. It is to be expected that differences in the environment, including differences in housing and husbandry practices and even in the methods used to record data, existed between institutions at any instance of time. In addition, it is likely that this changed *within* any given institution over time. This is why we made no distinction between accredited and nonaccredited zoos and why we made no distinction between different timeframes. It would be interesting to research potential differences in longevity and infant mortality between accredited versus non‐accredited zoos in follow‐up studies, for instance by zooming in on a more modern time span.

In our study, we did not focus on the direct effect of environmental variables on maximum longevity. For primates living in zoos, there are many examples of environmental factors that have been proven to influence animal welfare and overall health (Davey, 2007; Honess & Marin, 2006; Hosey, 2017; Mason, 2010, 2015; McKenzie et al., 1986; Wells, 2005; Zayan, 1991). Since we discovered continental differences in maximum longevity, it would be interesting to look deeper into the effect of environmental variables on the demography of zoo‐housed mangabeys, for instance by focusing on differing climatic conditions and caretaking methods. In addition, analyzing the DNA of zoo‐housed individuals by using molecular techniques could also provide more insights into the genetic background and health of the populations. This may help explain any existing regional differences in average lifespans and it may be of use in the context of breeding programs (Frankham et al., 2004).

## 5 CONCLUSIONS

To our knowledge, this is the first large‐scale study to explore the population biology and demography of zoo‐housed black crested and grey‐cheeked *(Lophocebus)* mangabeys. Our study shows that the females live on average longer than males, which is common in primates. Also, we show that the maximum longevity of the mother may serve as a proxy for the individual maximum longevity, likely due to a heritable component. Our results furthermore suggest that the maternal age at offspring birth negatively influences the survival of the offspring, which contradicts most literature on primate reproduction, and thus we believe that there must be an optimal breeding age for mothers. Lastly, we showed that, with juvenile mortality cases, infants died sooner when the parity of the mother was lower, but we found no effect of parity on maximum longevity in adults.

Both our models and survival analyses suggested that adult mangabeys housed in North American zoos live slightly longer than animals housed in European zoos. The causality of variation discovered in our study is impossible to infer from studbook data alone. Thus, more research is required. Still, the existence of potential regional differences highlighted by this overall study could improve upon existing intercontinental cooperation within the zoo community.

Our findings contribute to the general knowledge of (zoo‐housed) *Lophocebus* mangabeys, bring forth new research questions worthy of further study, and provide new insights for monitoring the health and management of the populations of these and potentially other, primate species.

## CONFLICTS OF INTEREST

The authors declare no conflicts of interest.

## ETHICS STATEMENT

This study was approved by GaiaZOO, as well as the EAZA Old World Monkey Taxon Advisory Group and the AZA Old World Monkey Taxon Advisory Group. We complied with the codes of ethics of the zoo(s) and associations involved. No animal experiments were performed to obtain any of the data; those were readily available.

## Supporting information

Supporting information.

## Data Availability

The data that support the findings of this study are available from the corresponding author upon reasonable request.
