# Supplementary material for: Maximum longevity and juvenile mortality in zoo‐housed mangabeys
Source: Zoo Biol. 2022 Apr 1;41(6):522–32. doi: 10.1002/zoo.21690 (PMC10084074; doi:10.1002/zoo.21690)
Supplement: Supplementary file 1 — Supporting information. [file ZOO-41-522-s001.docx]

**Supplementary Material**


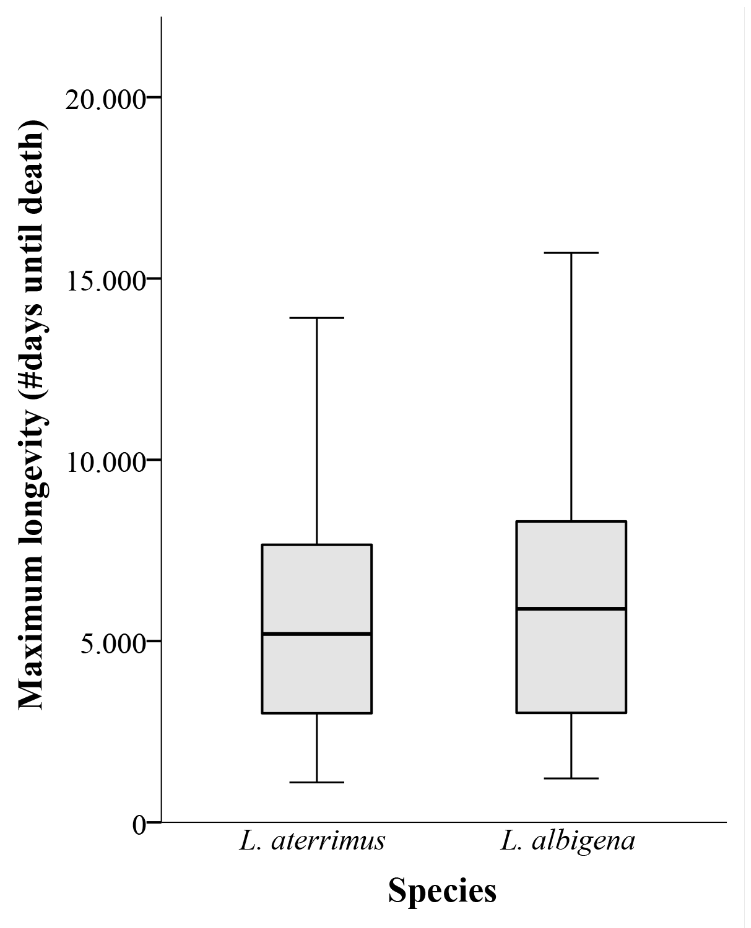
 **Figure I: The maximum longevity compared between *Lophocebus aterrimus* and *L. albigena*.** No significant difference exists between the maximum longevity of both species when looking at the historic population of adults (juvenile mortality cases were excluded, *L. aterrimus*: n=145, *L. albigena*: n=55).

**Table I**: Overview of all the variables that are investigated in the Generalized Linear Mixed Models (GLMMs). A definition is given per variable and if applicable, further explanation is provided on the meaning and/or extraction methods. Also the factor type and data type are provided. If the data was unavailable or unknown for certain individuals, no data were entered. Descriptive statistics (rounded off to one decimal) are provided as well. The international studbook (ISB) datasets served as the core information source. Population Management Software (PMx) was used to calculate F-adjusted. CI = Confidence Interval.

| **Variable [Factor type – Data type]** | **Brief definition** | **Further explanation** | **Mean (95% CI) [min-max] {sample sizes}** |
| --- | --- | --- | --- |
| **Dependent variables** | | | |
| Maximum Longevity [Target – Scale] | Life expectancy/ Maximum age in days. | Maximum longevity is also analyzed in Survival Analysis. For all analyses it is indicated whether juvenile mortality is in- or excluded. | All: 3,734.7 (3,425.3 – 4,044.2) [0.0 – 15,706.0] {n=519} |
| Juvenile mortality [Target – Nominal/Ordinal] | Death under certain infant age thresholds. | 1=Death within one day, 2=Death within one year, 3=Death within three years. The same as max. longevity, but focused on infants only. | 1.9 (1.8-2.0) [1.0 – 3.0] {n_1_= 71, n_2_=44, n_3_=55} |
| **Independent variables** | | | |
| Dead-lost-alive [Random – Nominal] | Whether the animal is dead, ‘lost to follow up’, or still alive. | 1=Dead, 2=Lost, or 3=Alive. If the animal is ‘lost to follow up’ we have no knowledge of whether it is currently alive or not. | 1.5 (1.4 – 1.6) [1.0 – 3.0] {n_1_=352, n_2_=69, n_3_=98} |
| Sex [Fixed – Binary] | The sex of the according individual. | 1=Male, 2=Female. | 1.5 (1.4 – 1.5) [1.0 – 2.0] {n_1_=249, n_2_=241} |
| Birth type [Fixed – Binary] | Whether the animal was captured, or born in a zoo. | 0=Wild born, 1=Zoo-born. | 0.8 (0.8 – 0.8) [0 – 1] {n_0_=88, n_1_=367} |
| Rearing [Fixed – Binary] | The way the individual was reared as an infant. | 0=Parent-reared, 1=Reared otherwise (the latter includes hand-reared and colony-reared individuals). | 0.1 (0.0 – 0.1) [0.0 – 1.0] {n_0_=346, n_1_=19} |
| Inbreeding [Fixed – Scale] | The inbreeding coefficient per individual (F-adjusted) | Even though the inbreeding is generally low in these populations, it is taken into account in Juvenile Mortality analyses. F-adjusted is used, which is calculated by PMx as:  F-adjusted = F * k + (1 – GD)*(1 – k), where GD is the Genetic Diversity and k is the proportion of the ancestry that is known. | 0.0 (0.0 – 0.0) [0.0 – 0.3] {n=438} |
| Father ID [Random – Nominal] | The identity of the father | Provided as ‘Sire’ by PMx. | {n=66 different fathers} |
| Father age at offspring birth [Fixed – Scale] | Age of the father in days when the individual was born. | Provided by PMx as ‘Sire Ages’ in days. | 4,312.1 (4,122.8 – 4,501.5) [1,066.0 – 11,486.0] {n=330} |
| Mother ID [Random – Nominal] | The identity of the mother | Provided as ‘Dam’ by PMx. | {n=86 different mothers} |
| Mother age at offspring birth [Fixed – Scale] | Age of the mother in days when the individual was born. | Provided by PMx as ‘Dam Ages’ in days. | 4,178.1 (3,992.9 – 4,363.4) [956.0 – 9,239.0] {n=336} |
| Mother parity [Fixed – Binary] | Whether the mother has had offspring before | 0=No previous offspring, 1=At least one previous offspring. | 0.7 (0.7 – 0.8) [0.0 – 1.0] {n_0_=87, n_1_=258} |
| Maternal maximum longevity [Fixed – Scale] | Life expectancy/ Maximum age of the mother in days | A potential proxy for 'mother quality'. Only interesting if the mother has deceased. | 7,829.7 (7,530.3 – 8,129.2) [2,462.0 – 14,004.0] {n=336} |
| Rearing type mother [Fixed – Binary] | The way the mother of the individual was reared | 0=Parent-reared, 1=Reared otherwise (the latter includes hand-reared, colony reared and foster reared individuals). | 0.0 (0.0 – 0.1) [0.0 – 1.0] {n_0_=328, n_1_=14} |
| Region born [Fixed – Nominal] | The region in which the animal was born. | 0=Europe, 1=North America, 2=Africa (wild born) | 0.5 (0.4 – 0.6) [0.0 – 2.0] {n_0_=312, n_1_=41, n_2_=90} |
| Region familiar [Fixed – Nominal] | The region the animal has lived most time of its life | 0=Europe, 1=North America, 2=Other (In Europe and North America there are only zoos. The ‘Other’ category includes individuals that lived mostly in the wild in Africa, as well as ~10 individuals that lived in Asian institutions). | 0.2 (0.2 – 0.3) [0.0 – 2.0] {n_0_=409, n_1_=81, n_2_=20} |
| Zoo born [Fixed – Nominal] | The zoo in which the animal was born. |  | {n=50 different zoos} |
| Zoo familiar [Fixed – Nominal] | The zoo in which the animal has lived the longest/the most. | This is a rough estimation, as certain animals changed zoos frequently. This reflects part of the ‘Region’ variable (Europe and North America), but in smaller categories | {n=72 different zoos} |

**Table II: Independent variables that influence maximum longevity and juvenile mortality (the dependent variables) as discovered in two different Generalized Linear Mixed Models (GLMMs).** The variables included in the Full Models (FM) and the Minimum Adequate Models (MAM) are given. For main effects of variables with a scale level of measurement, it is indicated whether the variable positively [+] or negatively [-] influenced the dependent variable according to the model (note that, in Model B, a ‘positive’ [+] impact meant the offspring survived significantly longer, and *vice versa*). MAM sample sizes are given and between squared brackets the percentage it covers of the total number of cases considered for processing (also provided). *p<0.05, **p=<0.01.

| FM variables | MAM variables and statistics | MAM sample size |  |
| --- | --- | --- | --- |
| Maximum Longevity (adults) - Model A: Parental variables | | |  |
| Fixed effects;  Father age at offspring birth, Mother age at offspring birth, Rearing type mother, Maternal maximum longevity, Mother parity Random effects;  Mother ID, Father ID, Dead lost alive | **Main Fixed effects;** [+] Maternal maximum longevity** (F=21.249, df1=1, p<0.001) [-] Mother age at offspring birth** (F=47.811, df1=1 p<0.001) | n=167 [47,9% of 349] **Notes:** Only adult animals (both alive and deceased) were considered. | FM AIC:  438,007 MAM AIC: 325,004   ΔAIC: 113,003 |
| Juvenile Mortality (infants) - Model B: Life history and parental variables | | |  |
| Fixed effects; Sex, Inbreeding, Zoo born, Father age at offspring birth, Mother age at offspring birth, Maternal maximum longevity, Mother # previous offspring Random effects; Mother ID, Father ID, Region Born | **Main Fixed effects;** [+] Maternal maximum longevity (F=2.925, df1=1, p=0.090) [-] Mother age at offspring birth* (F=5.317, df1=1, p=0.023) [+] Mother # previous offspring* (F=5.745, df1=1, p=0.018) | n=121 [79.6% of 152] **Notes:** Only infant animals (death before the age of three) were considered and divided into three categories (dead <2d, <366d, <1096d) | FM AIC: 1165,291 MAM: 1037,263   ΔAIC: 128,028 |
